# Multi-country case study on school health policy and its implementation in relation to COVID-19 control in Micronesia Small Islands Developing States

**DOI:** 10.1186/s41182-024-00590-8

**Published:** 2024-03-29

**Authors:** Fumiko Shibuya, Margaret Hattori-Uchima, Paul Dacanay, Florence Peter, Tarmau Terry Ngirmang, Rudelyn Dacanay, Rie Takeuchi, Calvin de los Reyes, Jun Kobayashi

**Affiliations:** 1 Department of Global Health, Graduate School of Health Sciences, University of the Ryukyus, 207 Uehara, Nishihara, Okinawa 903-0215 Japan; 2 Japanese Consortium for Global School Health Research, Nishihara, Japan; 3 School of Health, University of Guam, Mangilao, Guam; 4 Division of Health Sciences, College of Micronesia-FSM, Palikir, Pohnpei Federated States of Micronesia; 5 Women United Together Marshall Islands, Majuro, Marshall Islands; 6 Nursing Program, Palau Community College, Koror, Palau; 7 Faculty of Medicine, International University of Health and Welfare, Narita, Japan; 8 College of Arts and Sciences, University of the Philippines Manila, Manila, Philippines

**Keywords:** School health, Policy, COVID-19 control, Micronesia Small Islands Developing States

## Abstract

**Introduction:**

The COVID-19 pandemic caused school closures and rises in mental illness and non-communicable disease among school children worldwide. The Pacific Small Islands Developing States (SIDS) were also affected, but school health activities, which can effectively reduce negative effects of COVID-19, were not widely implemented compared to other Asia-Pacific countries. This study examined current school health implementation and related policies at national, local, and school levels in the Micronesia SIDS according to phases of COVID-19 control.

**Methods:**

Multi-country case study targeted the Federated States of Micronesia (FSM), Republic of the Marshall Islands (RMI), and Republic of Palau (ROP). These studies focused on school health implementation periods according to the PPR (Prevention, Preparedness, and Response) concept: Phase #1: prevention/preparedness, #2: early phase response, and #3: chronic phase response/recovery phase. Data were collected through policy document reviews that identified school health policies related to COVID-19 controls in the three phases and key informant interviews (KIIs) with 44 key informants (FSM, *n* = 14; RMI, *n* = 18; ROP, *n* = 12) whose work related to school health. The collected data were analyzed using content analysis methods according to the conceptual framework in this study.

**Results:**

This study identified three factors of school health implementation related to COVID-19 controls: promotion of decentralized education (FSM), implementation of COVID-19 controls in the school community (RMI), and disaster management for the protection of students including response to infectious disease (ROP). In Phase #1, no country had established a school health policy. In Phase #2, three enablers were identified in FSM and ROP, as reflected in COVID-19 controls by the education and health sectors. In Phase #3, RMI implemented COVID-19 controls in the school community. Documents on youth policy and disaster management in ROP were updated to reflect the chronic phase response and response to future public health crises.

**Conclusions:**

A decentralized education was instrumental in immediately implementing COVID-19 control measures in schools at national and local levels for coordination between education and health sectors. Despite each county’s multi-sectoral approach to engage COVID-19 controls in schools, local government organization requires strengthening and implementation of the formulated school health policy. In preparation for the next public health crisis, school health should be promoted that is integrated into both infection control and disaster management.

## Introduction

School health as defined by the American School Health Association is a service provided both within the school and the community that develops, implements, and evaluates support for school children [1]. Thus, school health services comprehensively cover such as factors a health education, physical education, and school lunches, and their implementation should be evaluated. This study aimed to examine factors that were enablers or barriers to the implementation of school health policies at national, local, and school levels in several Pacific Small Islands Developing States (SIDS) during the three phases of COVID-19 control. Furthermore, this study also aimed to compare the factors of school health policy and its implementation among the target countries and recommend the formulation of a common policy to accelerate school health in preparation for the next public health crisis.

The Health Promoting School (HPS) has been advocated by the World Health Organization (WHO) and other United Nations agencies to achieve healthy lifestyles and behaviors for school populations by developing supportive environments for health promotion [2]. The HPS paradigm is an institutional strategy designed to enhance health and academic achievement within school communities. It leverages the organizational capacity of schools to cultivate physical, socio-emotional, and psychological states that promote both health and positive educational outcomes [3]. The comprehensive approach conceptualized in the HPS for implementing school health has resulted in favorable health outcomes, including heightened physical activity, enhanced nutritional practices, and prevention of bullying [3, 4]. In terms of interventions to accelerate school health in Southeast Asia, Japanese organizations made efforts to strengthen school health promotion by disseminating the Hashimoto Initiative in 1998 [5]. The Hashimoto Initiative also helped establish an international partnership with several agencies to develop school health programs for global parasite control in 2000 [6]. In 2012, a program was initiated in South Asia to address school health and nutrition. The program extended the school health network in Asia and motivated participants to develop and implement school health policies [5].

The HPS has also been proposed in the Western Pacific region, but its implementation remains unclear in the Pacific Islands. Thus, case studies were conducted in a part of the Pacific Island region for further promotion of the HPS. As per a systematic review of school-based interventions in the Western Pacific in 2020, despite the limited number of eight school-based health interventions carried out in the region, their effectiveness was demonstrated in improving knowledge, attitudes, behaviors, and school health policies [7]. Approximately 235 million people, or about 20% of the world’s population, are adolescents living in the Western Pacific region [8]. Of this group, nearly 280,000 are between the ages of 5 and 29, including approximately 53,000 children between the ages of 5 and 14 [9]. In 2019, the Member States of the WHO Regional Committee for the Western Pacific reaffirmed their commitment to investing in the health of children and adolescents in the Asia-Pacific region by recognizing the seriousness of the health problems prevalent in the region [9]. To achieve this goal, they advocated a focus on school health, recognizing the interdependence of education and health. The regional framework was published by the WHO Regional Office for the Western Pacific in 2022, and it was proposed to implement this framework as a 5-year vision. Therefore, it is essential to implement the comprehensive approach to improve educational and health outcomes for children and adolescents, especially in large populations living in vulnerable regions where the adoption of the HPS has been limited.

The worldwide pandemic of coronavirus disease 2019 (COVID-19) due to the spread of SARS-CoV-2 has negatively influenced children and adolescents due to school closures. The public health emergency resulting from the COVID-19 pandemic has impacted the health of these children by affecting both their physical and mental conditions. According to a previous study, the abrupt cessation of school, social interactions, and extracurricular activities had a significant impact on children and adolescents, resulting in an increase in incidents of domestic violence and child abuse [10]. Both physical and social distancing measures, as well as seclusion and self-isolation, combined to influence an increase in risk factors for non-communicable diseases (NCDs) while the COVID-19 pandemic increased tobacco and alcohol use and decreased physical activity [11]. Both children and adolescents were most vulnerable to increased psychological distress, probably due to the need for greater social interactions [12]. To prevent the further spread of mutant strains of the virus in the future, the vaccination program for children and adolescents needs to be expanded, while strengthening existing activities and preparing for the impact of further school closures [13]. Therefore, an urgent recommendation was made to strengthen school health activities and preventive controls in response to the increasing risk of further school closures to manage the impact and control the spread of COVID-19.

An evaluation of school health implementation in the Pacific SIDS has not been conducted before or during the public health crisis of COVID-19. The SIDS are divided into three regions: Micronesia, Polynesia, and Melanesia, and each of the countries in these areas have responded to common challenges such as the prevention of NCDs and access to health facilities. Due to their small geographic size, remoteness, fragile environment, and lowered resilience to natural disasters, the Pacific SIDS face unique challenges. Further, the Pacific leaders declared a NCD epidemic in 2011 due to cross-cutting issues such as a changing lifestyle and increased tobacco use, and then the Ministers of Health of the Pacific Islands countries responded to it as a public health emergency [14]. The COVID-19 pandemic has significantly impacted SIDS, with approximately 28% of them being low-income countries [15], and inequities in the access to COVID-19 controls continue to make SIDS vulnerable to the pandemic [16]. SIDS in the Pacific have faced the potential devastation that could result from full exposure to the COVID-19 pandemic due to their small populations and fragile health services, most of these states could be severely affected within a short period of time [17, 18]. Micronesia, the smallest of the Pacific SIDS with the lowest population compared to Polynesia and Melanesia, is particularly vulnerable. The Micronesia SIDS member islands still face prevalent cross-cutting health issues among children and adolescents, including smoking, drug use, alcohol consumption, suicide, and teenage pregnancy [19–25]. In recent decades, there has been an extraordinarily high incidence of suicide among young males aged 15 to 24 in the Micronesia SIDS [26–29]. As part of the school health policy, these challenges recognize the need to address common cross-cutting issues among children and adolescents in the Pacific SIDS.

The implementation of school health faces several challenges, including differences in educational systems, cultural backgrounds, and financial issues. Education reform is influenced by political and fiscal perspectives and is classified into two education systems: centralized and decentralized. The concept of school-based management entails delegating decision-making authority from the national government to schools [30]. The United States adopted a decentralized education system based on the federal constitution to grant power over education to the states and local authorities [31, 32]. The education system in the Micronesia SIDS is also based on the United States model, and thus, the decentralized educational system is delivered via an educational organization [33–35]. Hence, the implementation of school health in the Micronesia SIDS context could be related to several factors and should be addressed at the administrative and school levels based on the education system of each country.

## Methods

### Study design and conceptual framework

**Fig. 1:**
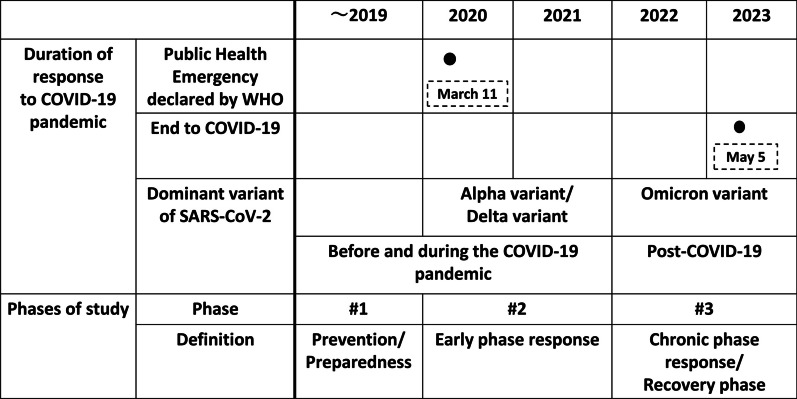
Phases used in this study set based on PPR (prevention, preparedness, and response)

This study used a multiple case study design to examine the factors of school health implementation through policy document reviews and key informant interviews (KIIs) because a case study explores a contemporary phenomenon in the current context and can include single or multiple cases to compare a different or similar situation [36, 37]. This study focused on the three phases of school health policy and its implementation: Phases #1 and #2, before and during the COVID-19 pandemic, respectively, and Phase #3, post-COVID-19 (Fig. 1). These phases were classified based on the epidemic period of the SARS-CoV variant because each variant expanded during different periods in each country. Furthermore, a conceptual framework was developed to compare school health policy and its implementation from multiple countries according to the concepts of PPR (Prevention, Preparedness, and Response), which has been advocated by WHO [38, 39]. In the conceptual framework, Phase #1 is defined as the period of prevention/preparedness, Phase #2 as the period of the early phase response, which encompassed the Alpha and Delta variants, and Phase #3 as the period of the chronic phase response/recovery phase, which encompassed the Omicron variant. Thus, the study combined policy document reviews and KIIs to triangulate possible convergence of the collected data from different data sources and to determine the consistency of the findings throughout each phase from multiple countries.

### Study setting and characteristics of the target countries

The study was carried out in three countries of the Micronesia SIDS: the Federated States of Micronesia (FSM), the Republic of the Marshall Islands (RMI), and the Republic of Palau (ROP). These three target countries of the Micronesia SIDS have overlapping issues relating to geography and difficulty in delivering education and health services. Table 1 shows the characteristics of the three countries such as political type, education systems, dominant variant of SARS-CoV-2, and period of the notification of school closure. This information summarized in Table 1 was collected from newspapers and archival sources and provides a reference to sort the timeline of the data sources.

**Table 1:** Characteristics of the three countries in relation to COVID-19 controls

Characteristics		Target countries		
		Federated States of Micronesia (FSM)	Republic of the Marshall Islands (RMI)	Republic of Palau (ROP)
Income group		Lower middle income	Upper middle income	Upper middle income
Political type		Decentralization	Decentralization	Decentralization
Education system		Decentralized system	Decentralized system	Decentralized system
Period of declaration of public health emergency		2020/01/31–2022/05/31	2021/02/18–2022/09/30	2020/03/17–2021/02/10
Period of country border closure		2020/03–2022/07/31	2020/01/24–2022/09/30	2020/03–2021/04
Start date of vaccination for SARS-CoV-2		2021/01/04	2020/12/29	2021/01
First case in the country		2021/01/08	2022/08/08	2021/05/31
Public notice for dominant variant of SARS-CoV-2				
Alpha variant				
Delta variant		2021/09/16		
Omicron variant		2021/12/01 (Pohnpei State Government)		
Period of official notification for school closure		2020/03-2022/08	2022/08/08-2022/09/12 (Private schools), 2022/08/08-2022/09/19 (Public schools)	2020/03/23–2020/07/31

This summary is according to the “World Bank’s Classification of Countries by Income” published by the World Bank [40] and references [45–47], and the information related to the COVID-19 controls was collected from newspapers and archival sources

The first country studied is the FSM, a country spread across the Western Pacific Ocean that comprises the four states of Pohnpei, Chuuk, Kosrae, and Yap. The country borders of FSM were closed between March 2020, and July 31, 2022, according to a declaration of public health emergency. The state government directed the public to prevent the spread of COVID-19 in each state, and school closures were also implemented at the state level according to the national government statement.

The second country is the RMI, an archipelagic island country consisting of 29 atolls and five islands. It was the region in Micronesia most influenced by the United States (US) after World War II due to its location. In RMI, vaccination against SARS-CoV-2 started on December 29, 2020, and it was advanced compared with the other countries. Also, the first case of COVID-19 occurred in August 2022, 1 month before the border was opened.

As the third studied nation, ROP is located in the North Pacific Ocean and consists of six island groups within the Caroline Islands chain. The country is divided into 16 states. In the ROP, a public health emergency was declared on March 17, 2020, although the Ministry of Education (MOE) published an official notification of school closure on March 23, 2020, after this declaration.

These target countries were selected according to the level of the World Bank’s Classification of Countries by Income [40] because this study aimed to recommend the formulation of a standard policy for the promotion of school health in the Pacific SIDS to prepare for the next public health crisis in the future. Thus, this study focused on these three countries with different economic situations to examine adaption of the policy to the whole Pacific SIDS and to extract the different contexts of these three countries.

### Data collection

#### Policy document review

A policy document review was conducted to describe the elements of existing school health before and during the COVID-19 pandemic. The main purpose of policy document reviews is to support and enhance evidence gathered from other data sources, as they play an important role in any data collection process when conducting a case study [37]. Relevant documents were extracted based on policy definitions advocated by the Centers for Disease Control and Prevention (CDC) [41]. The WHO declared COVID-19 a pandemic on March 11, 2020. Thus, we focused on investigating changes in school health implementation before and during the COVID-19 pandemic (Phases #1, #2), and post-COVID-19 (Phase #3) as the periods for policy document reviews [42]. The reviewed policy documents relating to school health implementation were sorted according to their classification (Table 2).

**Table 2:** Summary of the reviewed policy documents throughout Phases #1–#3

	Type of document		Country		
			FSM	RMI	ROP
			Title of document	Title of document	Title of document
	Memorandum of understanding		None	None	MOU of School Health Program Letter of MOH and MOE
Phase #1	Law	Education	Education Title 40, Chapter 1, § 101 Policy and purposes	Marshall Islands Public School System Act 2013	Education Title 22, Chapter 1 Education System, § 101 Declaration of policy and purposes
		Health	Title 41, PUBLIC HEALTH, SAFETY & WELFARE	Republic of the Marshall Islands Tobacco Control Act 2006 Public Health, Safety and Welfare Act, TITLE 7—PUBLIC HEALTH, SAFETY AND WELFARE	
		Other	Strategic Development Plan (2004–2023)		
	Policy	Education			
		Health		National Reproductive Health Policy and Strategy 2016–2018	
		Other	Nation Wide Integrated Disaster Risk Management and Climate Change Policy 2013		Palau National Youth Policy 2005 (First edition)
					Palau National Youth Policy 2016–2021 (Second edition)
	Regulation	Education		PUBLIC SCHOOL SYSTEM RULES AND REGULATIONS, TITLE 14 MINISTRY OF EDUCATION CHAPTER 13 HEALTH AND SAFETY	
		Health			
		Other			
	Strategy/plan	Education	FSM Association of Chief State School Officers (FACSSO) Resolution endorsing WASH in Schools	Multisectoral Early Childhood Development Project, INTERNATIONAL DEVELOPMENT ASSOCIATION PROJECT APPRAISAL DOCUMENT	
			Pohnpei State Strategic Development Plan		
			Chuuk State Strategic Plan for Education 2007–2012		
		Health	NATIONAL STRATEGIC PLAN OF ACTION FOR THE PREVENTION AND CONTROL OF NON-COMMUNICABLE DISEASES IN THE FEDERATED STATES OF MICRONESIA 2019–2024	WHO proMIND: profiles on mental health in development: Republic of the Marshall Islands	Republic of Palau Non-communicable Disease Prevention and Control Strategic Plan of Action 2015–2020 Healthy Communities Healthy Palau
				‘RMI NCD CRISIS RESPONSE PLAN’ NCD EMERGENCY RESPONSE TOWARDS A HEALTHY RMI Action Plan 2013–2018	
				3 Year Rolling Strategic Plan, October 2017–September 2019	
		Other		Republic of the Marshall Islands Joint National Action Plan for Climate Change Adaptation & Disaster Risk Management 2014–2018	National Disaster Risk Management Framework 2010
				National Strategic Plan 2020–2030	
	Administrative action/notification	Education			MOE COVID-19 Response: Directive No. 01-20
					MOE COVID-19 Response: Directive No. 02-20
		Health			
		Other			
	Guideline (manual)	Education			School Handbook (Second edition published 2019)
		Health			
		Other			Disaster Management Reference Handbook 2020
Phase #2	Law	Education			
		Health			
		Other			
	Policy	Education			
		Health			
		Other			MEMORANDUM MCCA CORONAVIRUS (COVID-19) RESPONSE MCCA DIRECTIVE NO. 01–20
	Regulation	Education			
		Health			
		Other			
	Strategy/plan	Education	Education Sector Strategic Development Plan 2020–2024		Planned action for the closing out of the current school year 2020
		Health			
		Other			
	Administrative action/notification	Education			
		Health	Position Description-School Pandemic Coordinator—Chuuk, Pohnpei, Yap, Kosrae, National: Five Temporary Positions for COVID-19 Response		DIRECTIVE NO. 189-20 BELAU NATIONAL HOSPITAL AND PUBLIC HEALTH MEASURES IN RESPONSE TO COVID-19
		Other			
	Guideline (manual)	Education	COVID-19 Standard Operating Procedure (SOP) SY: 2022–2023		Guidelines for Reopening of School: Directive No. 04-20
		Health			
		Other			
Phase #3	Law	Education			
		Health			
		Other			
	Policy	Education			
		Health			
		Other			Palau National Youth Policy 2022–2027 (Third edition)
	Regulation	Education			
		Health			
		Other			
	Strategy/plan	Education			
		Health			
		Other			
	Administrative action/notification	Education			
		Health			
		Other			
	Guideline (manual)	Education		COVID-19 Health and Safety Guidance for K-12 2022–2023	
		Health			
		Other			Disaster Management Reference Handbook 2023

*FSM* Federated States of Micronesia, *RMI* Republic of the Marshall Islands, *ROP* Republic of Palau

#### Key informant interviews (KIIs)

The KIIs were conducted to investigate the school health policy and its implementation from the perspective of key informants who implement school health activities. The key informants in each country are listed as participants from the national, local, and school levels and were requested to attend the interviews through snowball sampling (Table 3). In addition, the interview guide used an organizational model of the school health service, as proposed by the WHO [3], to explore the enablers and barriers involved in implementing school health activities [43] (Table 4). The interviews were conducted from the point of view of three timelines: current situation, past progress, and future vision. The interviews were undertaken between November 2022 and February 2023. KIIs were conducted face-to-face and online (Zoom meeting) with written and verbal consent obtained from the key informants. Each interview lasted approximately 60 min and was conducted by the principal investigator.

**Table 3:** Number of study participants and type of respondents at each level in the three countries

Implementation level	Type of respondents	FSM	RMI	ROP
National	Ministry/National Department of Education	1	2	2
	Ministry/National Department of Health	1	6	2
	Ministry of Human Resources, Culture, Tourism and Development			2
	Private sector		6	
Local	Department of Education	2		
	Department of Health	6		
	State Government			1
School	Public Primary School/Public Elementary School	2	2	2
	Public Secondary School/Public High School		1	2
	Private Primary School/Private Elementary School			1
	Private Secondary School/Private High School	2	1	
Total number of study participants (*n* = 44)		14	18	12

*FSM* Federated States of Micronesia, *RMI* Republic of the Marshall Islands, *ROP* Republic of Palau

**Table 4:** Interview questions posed to key informants from the point of view of the three timelines

No.	Timelines	Questions
1	Current situation	What do you think about the health issues or challenges among school children?
2		Who is responsible for your organization (school) in the implementation of school health?
3		What is the role of your organization (school) and community in the implementation of school health?
4	Past progress	How has your organization (school) contributed to students in school during the COVID-19 pandemic?
5		What factors promoted the implementation of school health or health education?
6	Future vision	How do you want to improve the current situation in the implementation of school health in the future?

### Data analysis

**Fig. 2:**
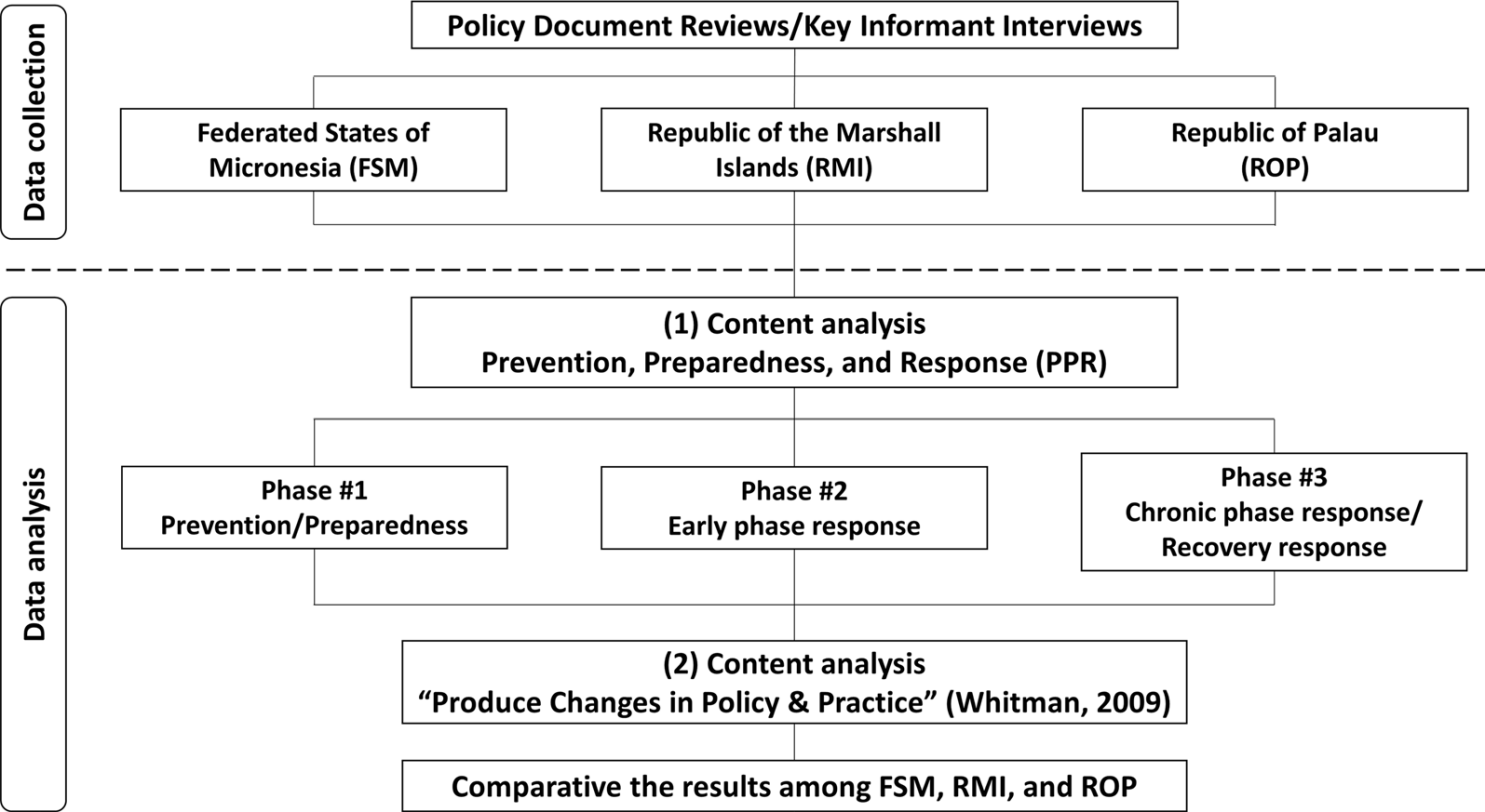
Flowchart of the multi-country case study

The collected data were analyzed using content analysis methods according to the two conceptual frameworks of PPR (Prevention, Preparedness, and Response) and “Produce Changes in Policy & Practice” developed by Whitman in 2009 (Fig. 2) [44]. The framework developed by Whitman was utilized due to its significant role in successful global school health implementation. It provides a comprehensive structure that includes the following 12 major factors contributing to successful policy implementation: (1) Vision and Concept/International and National Guidelines; (2) Dedicated Time and Resources; (3) Stakeholder Ownership and Participation; (4) Team Training and Ongoing Coaching/Learning Community; (5) Cross-Sector Collaboration; (6) Champions and Leaders at All Levels; (7) Data-Driven Planning and Decision-Making; (8) Administrative and Management Support, (9) Adapting to Local Concerns; (10) Attention to External Forces; (11) Critical Mass and Supportive Norms; and (12) Stage of Readiness. Each conceptual framework was used to adapt both the data collection process and data analysis to the objectives and justification of the study.

As the first procedure in the data analysis, the extracted policy documents and interview data were analyzed throughout the three phases to classify the publication periods according to the framework of the PPR. In the second procedure, interview data were assorted based on the 12 components in the “Produce Changes in Policy & Practice” and the factors related to school health policy and its implementation were extracted.

## Results

This study examined the factors reflecting the context of school health implementation in each country and identified its status throughout the target pre-pandemic and pandemic response phases of the COVID-19 controls. From the deductive content analysis, the results of which were compared with the conceptual framework of “Produce Changes in Policy & Practice” [44] in Table 5, this study identified the three novel factors of school health implementation related to COVID-19 controls: Phase #1: promotion of decentralized education (FSM), Phase #3: implementation of COVID-19 controls in the school community (RMI), and Phase #3: disaster management for the protection of students including response to infectious disease (ROP). These three novel factors did not fit within this conceptual framework and thus were recognized as novel findings of the present study. In Phase #1, commonalities indicated that no school health policy had been established in each country. In Phase #2, three enablers were identified in FSM and ROP, as reflected in COVID-19 controls by both the education and health sectors. RMI implemented COVID-19 controls in the school community in Phase #3. In ROP, documents on youth policy and disaster management were updated to reflect the context of the chronic phase response and response to future public health crises. Table 6 shows that school health programs/activities were implemented under the involvement of multiple sectors and stakeholders at the national, local, and school levels in each country.

**Table 5:** Characteristics of factors involved in implementing school health policies in the three countries

“Produce changes in policy & practice” (Whitman, 2009)		Extracted factors		
No.	Name of factor	FSM	RMI	ROP
1	Vision and concept/international guidelines	#1: Lack of school health policy (−)	#1: Lack of school health policy (−)	#1: Lack of school health policy (−)
				#1: Memorandum of Understanding of School Health Program (+)
2	Dedicated time and resources			
3	Stakeholder ownership and participation			
4	Team training and ongoing coaching/learning community	#2: Effect of establishing a manual for COVID-19 controls at schools (+)		#2: Effect of establishing a manual for COVID-19 controls at schools (+)
5	Cross-sector collaboration	#2: Cooperation between the education and health sectors (+)		#1: Multi-sectoral approach in implementing youth policy (+)
				#2: Cooperation between the education and health sectors (+)
6	Champions and leaders at all levels			
7	Data-driven planning and decision-making			#3: Youth policy focusing on specific health issues (+)
8	Administrative and management support	#2: Administrative and management support for COVID-19 controls (+)		#2: Administrative and management support for COVID-19 controls (+)
9	Adapting to local concerns			
10	Attention to external forces			
11	Critical mass and supportive norms			
12	Stage of readiness			
Novel factors of the study		#1: Promotion of decentralized education (+)	#3: Implementation of COVID-19 controls in the school community (+)	#3: Disaster management for the protection of students including response to infectious disease (+)

FSM: Federated States of Micronesia; RMI: Republic of the Marshall Islands; ROP: Republic of Palau; #1, #2, #3: Phase #1, Phase #2, Phase #3; +: enabler (positive factor); −: barrier (negative factor)

**Table 6:** Stakeholders of school health at each implementation level

Implementation level	FSM	RMI	ROP
National level	National Department of Education	Ministry of Education Sports and Training, Public School System	Ministry of Education
	National Department of Health and Social Affairs	Ministry of Health and Human Services	Ministry of Health and Human Services
			Ministry of Human Resources, Culture, Tourism and Development
Local level	Department of Education in each state (Pohnpei, Chuuk, Kosrae, and Yap)	NA	State Government Department
	Department of Health and Social Affairs in each state (Pohnpei, Chuuk, Kosrae, and Yap)		
School level	Private sectors (UNICEF, UNFPA, Red Cross)	Private sectors (Youth to Youth in Health, Women United Together Marshall Islands, UNICEF, UNFPA, Red Cross)	Private sector (UNICEF), Japanese Government, JICA

*FSM* Federated States of Micronesia, *RMI* Republic of the Marshall Islands, *ROP* Republic of Palau, *NA* not applicable, *UNICEF* United Nations Children's Fund, *UNFPA* United Nations Population Fund, *JICA* Japan International Cooperation Agency

### Phase #1 prevention/preparedness

One barrier was identified through Phase #1 prevention/preparedness, namely lack of school health policy (FSM, RMI, and ROP). Further, three enablers were extracted: promotion of decentralized education (FSM), Memorandum of Understanding of School Health Program (ROP), and multi-sectoral approach in implementing youth policy (ROP).

### Vision and concept/international guidelines

#### Lack of school health policy (FSM, RMI, and ROP)

The three countries have commonality in the establishment of a fundamental law addressing education; however, the law did not include relevant content on school health. There was no legally established school health policy in the three countries as confirmed by the MOE and Department of Education (DOE) in each country. Responses of the key informants shown in italics help to provide evidence on how implementation in each country actually improved or promoted school health implementation during and after the COVID-19 pandemic.

*I do know that each State Department of Education, do work with the health and with parents’ communities to make sure that the kids who are in the school system are healthy. However, I think there is still a lack of written policies, as far as you know, in this area of health. (FSM-National DOE [NDOE])*

*We don't have a school health policy in the schools. The curriculum right now does not allow us to have sustainable discussions around health. We talked to the curriculum programs at the public school system about institutionalizing policies to address some of these issues on teen pregnancy, suicide, NCDs, and communicable diseases, but it hasn't really connected. (RMI-Ministry of Health and Human Services [MOHHS])*

*We do not have a school health policy between MOE and MOH, we have processes on how we do work based on the MOU [Memorandum of Understanding] of the School Health Program. (ROP-MOHHS)*

#### Promotion of decentralized education (FSM)

Decentralization is reflected in the formulated policies reflecting the diverse visions and cultures, and different policies have been implemented in the three countries within the same region of Micronesia (Table 2). Only the federal state of FSM has adopted a decentralized education system, which was then included in the fundamental law of education. The FSM National Government DOE established a fundamental law of education (*Education Title 40, Chapter 1, § 101 Policy and purposes*) that promotes a decentralized education system in the four states of Pohnpei, Chuuk, Kosrae, and Yap.

This education law is declared as follows: …*to be the policy of the FSM to provide for a decentralized education system in FSM which shall enable the citizens of the FSM to participate fully in the development of the islands as well as to become familiar with the Pacific community and the world* [45]. The extracted enabler related to decentralized education system from FSM was reflected in the provision of education at the state level. Also, the Pohnpei State DOE, which is one of the state government departments, established and implemented a Fundamental Law of Education at the state level in accordance with the national version of the law.

*I thought that's the way it should be the National Department of Education should establish all of those then the states could be the ones implementing those. (FSM-NDOE)*

In contrast, RMI and ROP have established their fundamental law of education at the national level. This context of a federal state in FSM is reflected in the difference in the education system, with FSM strongly promoting implementation of decentralized education compared with RMI and ROP.

### Vision and concept/international guidelines

#### Memorandum of understanding of school health program (ROP)

A MOU regarding the school health program letter in ROP was published between the MOE and the MOH in 2004 [46]. Both the MOE and MOH have cooperated to conduct health examinations such as physical and oral health assessments at schools, and an immunization program has also been provided to the students based on this MOU. This MOU has also contributed to providing not only physical health checkups but also psychological checkups by supplying school counselors and monitoring conditions of mental illness among school children at both the primary and secondary levels of public school.

*It's a memorandum of understanding about school health programs between the Ministry of Health and the Ministry of Education. We can have some sort of agreement and understanding on how we provide the services within the school environment. (ROP-MOHHS)*

### Cross-sector collaboration

#### Multi-sectoral approach in implementing youth policy (ROP)

The enabler in ROP during Phase #1 shows the multi-sectoral approach practiced in ROP and its implementation by not only the health and education sectors, but also relevant sectors that have contributed to solving cross-cutting health issues among school children. After the publication of the MOU of School Health Program, the “*Palau National Youth Policy*” was issued by the Ministry of Community and Cultural Affairs in 2005 to encourage youth activities including health programs. This youth policy implemented cross-cutting between multiple sectors of health and education and included specific health challenges to prevent NCDs and active sexual behavior among youth in Palau.

*Locally, we have one of the ministries of the Government (Ministry of Community and Cultural Affairs) is tasked with making a policy for youth (Palau National Youth Policy). I think that its policies can play a really critical role in the improvement of school health policies. Although the youth policy is a much broader policy, it's about youth. (ROP-MOHHS)*

### Phase #2 early phase response

In Phase #2 early phase response, FSM and RMI appeared to have positive factors in common. Three enablers were identified: cooperation between the education and health sectors, administrative and management support for COVID-19 controls, and effect of establishing a manual for COVID-19 controls at schools.

### Cross-sector collaboration

#### Cooperation between the education and health sectors (FSM and ROP)

In ROP, the Minister of Education provided a strategy (*Planned action for the closing out of the current school year 2020*) showing the scenarios for COVID-19 measures at schools. The scenarios were *(1) if no COVID-19 case occurs in Palau* and *(2) in the direction of President, the MOH, and the National Emergency Committee or if a COVID-19 case occurs anytime before the end of the school year*. The administrative order relating to the COVID-19 response (*MOE COVID-19 Response: Directive No. 01-20/No. 02-20*) that was provided during Phase #1 was implemented at the both the elementary and high school levels of public schools. The school principal at the public elementary school mentioned that a school manual was developed according to the administrative orders.

In FSM, the National Government DOE issued a strategy (*Education Sector Strategic Development Plan 2020–2024*) to respond to COVID-19 from both the National and the State DOE but did not include measures for school closure during the COVID-19 pandemic. This strategy aimed to minimize the disruption and negative impact of COVID-19 on educational outcomes and the overall operation of the education sector in FSM.

*We have a partnership with our Department of Health and Social Services whereby in cases where we will need immediate help in the area of health situations for the kids. Then we will ask them to come in and assess situations on the ground in the State Department of Education. Health specialists will also be working with them in scheduling activities that they can come in and do on our campuses like health screening for the kids, not just because the health specialist is responsible for all grade levels. (FSM-DOE)*

*Actually, a school health program is now running, doesn't have a specific one (school health policy). I think what really helped to promote the school health and a good working relationship with the Ministry of Health and the Ministry of Education. (ROP-MOHHS)*

### Administrative and management support

#### Administrative and management support for COVID-19 controls (FSM and ROP)

In FSM, an administrative order was issued (*Position Description-School Pandemic Coordinator—Chuuk, Pohnpei, Yap, Kosrae, National: Five Temporary Positions for COVID-19 Response*) by the FSM National Government Department of Health and Social Affairs (DHSA). This order aimed to hire “School Pandemic Coordinators” to assist the DHSA in supporting the Education School Systems to deal with expectations for COVID-19 preparedness from the time the schools were opened in the Fall of 2021 onward. The primary responsibility of the School Pandemic Coordinators involved facilitating the formulation and implementation of health and safety plans, which articulate clear mitigation strategies to enable schools to safely conduct their activities. Additionally, the coordinator was expected to act as a liaison between the FSM DHSA and educational or school authorities to ensure the successful implementation of activities and mandates. In fact, the coordinator in the Pohnpei State DHSA contributed to delivering hygiene materials and personal protective equipment (PPE) to lead in the prevention and treatment of COVID-19 at schools. These donations were supplied by private sector agencies such as UNICEF and the Red Cross, and the School Pandemic Coordinator coordinated the provision of the donations at each school. Aside from the School Pandemic Coordinator, the “School Health Coordinator” is responsible for immunization and has monitored SARS-CoV vaccinations during the COVID-19 and chronic phases at schools. Moreover, almost all respondents acknowledged that the role of both coordinators was quite important in promoting school health activities in response to the pandemic.

The ROP implemented an administrative directive (*DIRECTIVE NO. 189-20 BELAU NATIONAL HOSPITAL AND PUBLIC HEALTH MEASURES IN RESPONSE TO COVID-19*) outlining procedures for reporting COVID-19 cases. This initiative involved collaboration between the MOH and the National Emergency Committee to ensure the establishment and maintenance of suitable quarantine, isolation, and alternative care facilities for individuals diagnosed as having COVID-19, their close contacts, and travelers, among other responsibilities. This notification addressed the response to COVID-19 at schools and the notification of learning methods and continuity of operations and contingency planning during the pandemic response.

*There is a certain standard of procedure that education has given them, like within these schools, during the COVID-19. They have to have, for example, an isolation area in their schools. Basically, what I do is this fund that I'm working on, was given to us by the CDC. It's a reopening grant describe this given to us to help the schools of Pohnpei kind of help them open safely or continue operating safely under or during the COVID-19. I would just go to the schools and meet their staff, principals, and vice principals and kind of get the needs for the school mostly like infection prevention staff that they need, like hygiene, for example, the ice or the cleaning supplies for their isolation area and their beds. (FSM-Department of Health and Social Affairs [DOHSA])*

*Some of those COVID-19 preventive measures are still applicable to ensure that there's no spread of communicable diseases on the school campus during the school year. Preventive measures were issued to all school stakeholders, chairs, school principals, and parents to guide us on how we manage schools during those COVID-19 years. (ROP-Ministry of Education [MOE])*

### Team training and ongoing coaching/learning community

#### Effect of establishing a manual for COVID-19 controls at schools (FSM and ROP)

A manual on COVID-19 controls at schools was issued by the education sector in FSM and ROP and appeared to copy the administrative order for these countries at the national level. In FSM, the manual for COVID-19 controls at schools (*COVID-19 Standard Operating Procedure (SOP) SY: 2022–2023*) was provided by the Pohnpei State DOE and was implemented at the state level according to the SOP. This manual was issued by the Pohnpei State DOE to manage the COVID-19 measures at schools supported by the Pohnpei State DHSA. The Pohnpei State DOE mentioned that both the education and health departments in Pohnpei State cooperated to manage school closure and school reopening.

ROP issued the *Guidelines for Reopening of School: Directive No. 04-20*, which implemented COVID-19 measures at schools according to the manual that defined reopening of schools and COVID-19 preventive measures. Also, the MOE collaborated with the National Emergency Committee and the MOH, and this guideline was shown at the reopening of schools, with implementation required by students, teachers, school staff, and other school stakeholders. The school principal at the Koror Public Elementary School reported that the “*Standard of Practice for Re-Opening of School: Effective Immediately*” was developed to show students the rules pertaining to COVID-19 measures and class schedules when teachers monitored the reopening of school. Also, the school principal at the Palau Public High School mentioned that it provided a letter to parents that described the reopening of school after school closure and the measures taken to prevent COVID-19 at schools.

In RMI, no manual or guidelines for COVID-19 at schools were issued during Phase #2. The period of the COVID-19 pandemic and border closure was shorter compared with those in the other two countries, and therefore, the period of school closure also was shorter in RMI as mentioned by the public school system.

*With each of the states, I think I'm trying to think about what we call whether we work with them for their standard operating procedures during the pandemic and after the pandemic. There are always consultations between the national and the state with regard to anything that has to do with education and especially during the pandemic, we made sure children are protected. And so there were interim policies that were developed to address the pandemic. (FSM-NDOE)*

*I think there are lessons learned from our experience with COVID-19 on how we can better protect our children in schools. How we can train our teachers and school personnel on how to apply preventive measures in the school environment makes. We don't know when COVID-19 is going so it's important for us to continue to refresh the skills and the training and then to continue testing if they need to. (ROP-MOHHS)*

### Phase #3 chronic phase response/recovery phase

In Phase #3 chronic phase response/recovery phase, the diversity of the factors was identified in RMI and ROP. Because the period of dominance of COVID-19 was different in each country, the enablers also seemed to be different. In RMI, one enabler contributed to implementing the COVID-19 controls in the school community. In ROP, two enablers were promoted to prepare for the next public health crisis from the lesson learned of the COVID-19 pandemic: youth policy focusing on specific health issues and disaster management for the protection of students including response to infectious disease.

#### Implementation of COVID-19 controls in the school community (RMI)

In RMI, there has been a trend toward school decentralization through the implementation of Community-Based Governance of Schools. Schools in Majuro, Kwajalein, and some outer islands have been decentralized and are now under local government control [47]. During Phase #3, RMI issued a guideline (*COVID-19 Health and Safety Guidance for K-12 2022–2023*) by the Ministry of Education Sports and Training, Public School System. The aim of this guidance is to reduce the transmission of COVID-19 within the school community through mitigation, minimization, handling, and supervision. The guidelines for this work were based on the National Disaster Committee’s COVID-19 Community Level guidelines and were further refined using the Ministry of Health and Human Services (MOHHS) recommendations for schools, operational guidance provided by the CDC for educational institutions from kindergarten to twelfth grade, and early care and education programs, which were designed to facilitate secure in-person learning. The Ministry of Education, Sports and Training indicated that school closure would be implemented for a while and then students would start doing take-home studies during the COVID-19 pandemic. The MOHHS reported that this guidance for the school reopening project was part of a large partnership through a US CDC grant, which was basically provided to institute testing in the schools. The implementation of this guidance was evaluated in terms of supply, policies, training in place, and financial support for providing a school health nurse.

*COVID-19 didn't invade all the islands. Only a few schools on the outer island were closed. Those were the schools that we concentrate on the rise of the schools was still open. Because COVID-19 didn't enter their border, so the schools were not. We're still COVID-19 free and we asked teachers to continue teaching until they reported to it that there are cases of COVID-19, then that's when we started working with someone on the learning package. (RMI- Ministry of Education Sports and Training, Public School System)*

### Data-driven planning and decision-making

#### Youth policy focusing on specific health issues (ROP)

The *Palau National Youth Policy* was updated to indicate a common vision and objectives for youth development by the Ministry of Human Resources, Culture, Tourism and Development, Division of Youth and Career Development. The former *Palau National Youth Policy 2016–2021* (second edition) included a comprehensive review of the situation of youth and prioritized “Health and healthy lifestyles”. The youth policy recognizes that supporting youth development requires a multi-sector approach to issues concerning youth that cuts across different sectoral mandates, and it is crucial that key agencies work together effectively to promote development among the youth of Palau.

An updated *Palau National Youth Policy 2022–2027* (third edition) considered the continued relevance of the policy’s priorities and objectives taking into account more recent data and feedback from youth and key stakeholders. The policy priority objective is to ensure that all youth in Palau achieve the highest possible level of health. This is facilitated through their access to high-quality, youth-friendly programs, health information, services, and support, alongside the presence of robust and supportive families and communities. To accomplish this objective, the existing health issues among youth were reflected in the youth policy such as prevention of mental illness and drinking alcohol, increasing physical activity, and access to information on sexual and reproductive health and rights.

*We did the youth, like the consultation for the youth policy and we conducted a survey of the 151 participants from different high schools and they mentioned that the common issue that the use of alcohol and tobacco use these issues. (ROP- Ministry of Human Resources, Culture, Tourism and Development)*

*I think our first priority now is NCDs, particularly child obesity which has been identified for several years and it's still continuing to be an issue for our school children. Also, the issues about tobacco use or use drinking alcohol, teenage pregnancy, and suicide are used at we try to collect data through the YRBS (Youth Risk Behavior Survey) that we conduct every year. (ROP-MOE)*

#### Disaster management for the protection of students including response to infectious disease (ROP)

The National Emergency Management Office issued the *Disaster Management Reference Handbook*, which has addressed disaster risk reduction (DRR) in the education sector at the school level since Phase #3. The handbook emphasized the MOE as a key stakeholder and highlighted the role of schools as potential community gathering or evacuation sites. It also underscored the significance of integrating DRR concepts into the school curricula. The expectation is that the youth, once exposed to these concepts, will disseminate their newfound knowledge to their families. The MOE has shown its dedication to incorporating climate change and disaster risk management into the Grade 7–8 science curriculum in public schools. The MOE is supporting teachers by providing climate science training that aligns with the updated curriculum. The MOE actively engaged in governmental and community-driven planning initiatives related to state-level DRR Action and Evacuation Plans to incorporate the topics of climate change and disaster risk management into formal education. These plans assessed the suitability of designated school buildings to function as climate-resilient and disaster-proof evacuation shelters. In addition, this handbook included responses to communicable diseases such as dengue, tuberculosis, HIV, leprosy, and COVID-19. These communicable diseases, including COVID-19, have had the most impact and have required the most measures against infectious disease during and since Phase #3.

*I think the one thing that we have been doing and it's not just for the post but during COVID-19, we've really supported the school in terms of testing and education, and PPE. We distribute masks and sanitizers throughout all the schools. We've even trained some personnel on how to apply preventive measures and but a lot of the support that we have been outsourcing to the Ministry of Education is primarily for the prevention of COVID-19. (ROP-MOHHS)*

## Discussion

The present analysis showed that both enablers and barriers to school health policies and their implementation have been addressed in relation to COVID-19 controls at the national, local, and school levels among the target countries throughout Phase #1–#3. This study also confirmed the need to formulate school health policy based the lessons learned from the COVID-19 controls according to the concepts of disaster management including PPR and found that the commonality of the extracted factors can also support relevant decentralized education in the Pacific Islands.

Even in small island nations, different islands contain different ethnic groups, and political decentralization has been effective in mobilizing public finances. In Phase #1 prevention/preparedness, the barrier of “Lack of school health policy” was present in all three countries. The factor of “Promotion of decentralized education” in FSM was recognized to deliver control of education from the national to the state government. Thus, in the FSM, decentralized education has been implemented at the state level according to the Fundamental Law of Education advocated by the National Government. One of the commonalities in the school curriculum, its construction based on the US public school system education model, has been implemented in the three target countries. Decentralization is reflected in political and financial contexts and is defined as involvement in transferring decision-making and other responsibilities vested in a central authority to a local authority [32]. This case illustrates the primary role played by the decentralized education system.

Decentralized education might be adapted as an enabler of school health implementation due to decentralization in the Pacific Islands. In the FSM, school health policies related to the COVID-19 controls were implemented based on decentralization, which contributed not only to educational achievement but also to the improvement of health promotion. However, FSM is strongly multi-ethnic, and it could be difficult to integrate school health policies from the perspective of the differences in culture and health behavior among the four states. Tanzania, as another country practicing decentralization, has the potential to improve its health promotion services by having health sectors at the local level combine both their abilities and institutional capacities with their own authority [48]. Aside from its positive impact on the health sector, a decentralized school development plan can influence success in enhancing the attendance rate among school children [49]. A comparative study of Thailand and South Korea showed that both countries adopted policies at the central government and school levels that allow implementation of policy at the administrative (macro) level [50]. Decentralization has been implemented in Asia and African countries as evidenced by these previous studies, and the present study indicated that decentralization applied to the implementation of school health services can be similarly recommended in the Pacific Islands.

In previous studies in Lao PDR and Thailand, integration of these countries’ policies was recommended at each administrative level to enhance implementation of their respective national school health policies [51, 52]. The study conducted in Lao PDR in 2014 [51] found that policy implementation is affected by organizational factors across different administrative levels, ranging from national to individual schools. This suggested the development of a national plan with a clear long-term perspective and emphasized the significance of effective human resource management, including systematically organized training at each administrative level. Furthermore, the study conducted in Thailand in 2018 [52] suggested improvement of the implementation process of school health initiatives within Thailand’s decentralized education system and recommended alignment with the established educational strategies outlined in the National School Health Policy. Thus, as shown in the situation before the COVID-19 pandemic, the implementation of school health policies emphasized the importance of a decentralized education system at both the national and local levels.

In view of the need for coordination between the education and health sectors, decentralization has been instrumental in the immediate implementation of COVID-19 controls within schools at both the national and local levels in the target countries. During the COVID-19 pandemic (Phase #2), FSM and ROP advocated COVID-19 controls in the school setting that were published as an administrative action/notification. These commonalities among federate nations indicated that decentralization was well organized at the state level, and there was cooperation between the state and national governments. In RMI, however, the guideline on the COVID-19 measures to be taken at schools was published throughout Phase #3. Consequently, COVID-19 measures applied in the schools were managed according to the phase of the infection within each nation. A previous study in Ethiopia indicated that the obstacles affecting decentralized educational management are related to fundamental aspects of education and human resource development and that they should be constructed in a decentralized education system in a manner that is relevant to the system’s organizational capacity and stakeholders’ involvement [53]. Therefore, these extracted enablers should be considered for improved preparedness and should be addressed before the next public health crisis.

In preparation for the next public health crisis, pandemic PPR should be dealt with at the local level. The three target countries have published policies and plans related to disaster management, and they all involve the education sectors in implementing disaster risk management at schools during Phase #1 prevention/preparedness. Specifically, after the National Emergency Management Office in Palau issued their Disaster Management Reference Handbook, a reduction in disaster risk in the education sector at the school level has been shown since Phase #3 chronic phase response/recovery phase. To improve the effectiveness of PPR strategies, the various factors that contribute to the onset of a health crisis must be considered. This can be achieved through a comprehensive One Health approach, which goes beyond biological considerations [54]. The lack of such focus would indicate that an integrated and coordinated PPR strategy during the COVID-19 pandemic has not been attained [39]. Therefore, promotion of this approach should help to implement the PPR strategy at both national and local levels, including in the schools, and may address inclusive preparedness for future public health crises.

Although a multi-sectoral approach was implemented to engage control of COVID-19 at the schools in each country, to meet the next public health crisis, organization at local government should also be strengthened to implement school health measures according to the formulated policy. ROP has proceeded to respond to the post-COVID-19 period and future public health crises by protecting school students through the two extracted enablers of youth policy focusing on specific health issues and disaster management, including a response to infectious disease.

The youth policy has contributed not only to enhancing increased physical activities and decision-making but also to solving health matters among adolescents, such as the need for increased knowledge and skills to prevent mental illness, increased physical activity to reduce the NCDs, and increased access to information on sexual and reproductive health and rights. As the MOU on the school health program between the MOE and MOH has concluded, multiple sectors and the state government are now involved in promoting school health activities in ROP. A previous study evaluating the effectiveness of decentralization in Ethiopia reported improved performance in the public education and health sectors, significant improvement in the rising school enrollment rate in schools, and increased access to antenatal care among pregnant women [55]. These findings suggest that a multi-sectoral approach involving not only the education and health sectors but also other relevant sectors can enhance the cross-cutting health issues among adolescents.

It would be effective to strengthen PPR without creating separate PPR strategies for infectious disease control and disaster preparedness. DRR is one of the common challenges in the Pacific because most DRR strategies in Asia-Pacific have focused on natural disasters and provide only limited management of both biological hazards and emergencies [56]. In the post-COVID-19 Phase #3, RMI responded to the COVID-19 issues within the school community through the extracted enabler of “Implementation of COVID-19 controls in the school community”. The public school system, a part of the Ministry of Education, Sport, and Training in RMI, published “*COVID-19 Health and Safety School Guidance for K-12 School Year 2022–2023*” in 2022 during the Phase #3 chronic phase response/recovery phase. It was aimed at preventing, reducing, managing, and monitoring the transmission of COVID-19 and is based on the National Disaster Committee’s COVID-19 Community Level, a published guideline focused on providing assistance for safe schools according to the concepts of DRR. Likewise, the Framework for Resilient Development in the Pacific recognized that cross-cutting issues such as climate change and the risk of disasters be included and advocated involving the health sector as one of the key sectoral levels [56]. The Pacific region’s progress in DRR might strengthen the countries at national, local, school levels due to decentralization.

Strengthening the implementation of school health based on disaster management in the Pacific Islands should involve multiple sectors relevant to school health activities. Nevertheless, the Pacific Islands remain challenged to construct a school health system based on such policies, and this may be difficult due to geographic issues compared with continental countries. The Review of COVID-19 Disaster Risk Governance in Asia-Pacific stated that when considering integrated measures to handle the spread of infectious disease, DRR must be enhanced with several existing policies and governance in tandem with local (state) governance, as was done in the COVID-19 pandemic [56]. In 2011, WHO advocated strengthening of the disaster risk management system by integrating it into a national public health policy [57]. Previous studies have recognized the necessity of organizing holistic approaches to adapt responses to the spread of both infectious disease and disasters to respond efficiently to them [58, 59]. Therefore, it is essential to create a school health policy that promotes preparedness for future public health crises and effective disaster management, as learned from the experiences during the COVID-19 pandemic in the Pacific Islands.

Currently, there is very little organized and publicly available information on the status of the enactment of policy documents and implementation of school health in the Pacific Islands. The WHO Western Pacific Regional Office established the Regional Framework for School Health in 2022 to encourage the alignment of national policies for healthy schools [9]. However, the present study could not identify any relevant findings on the implementation of this regional framework, and thus, further evaluation of its implementation in the Pacific Islands will be required.

This study has two limitations that must be kept in mind when considering its findings. First, this study only examined factors in some of the remote islands. Specifically, KIIs in the FSM, which is a federation of states, were conducted only in Pohnpei State. Because the capital of FSM is in Pohnpei State, the authors decided to conduct fieldwork there in consideration of the geography of the nation. Consequently, the key informants who responded at the local (state) level included only those from Pohnpei State and not those from the other three states of Chuuk, Kosrae, and Yap. Second, the data could not be collected in a timely manner in line with PPR concepts during Phase #1–#3, and thus, data collection in these multi-country case studies was performed retrospectively.

## Conclusions

This study identified the three novel factors of school health implementation related to COVID-19 controls in the targeted countries: promotion of decentralized education in FSM, implementation of COVID-19 controls in the school community in RMI, and disaster management for the protection of students including response to infectious disease in ROP. The decentralized education in the target countries has been instrumental in the immediate implementation of COVID-19 control measures in schools at both the national and local levels because of the need for coordination between the education and health sectors. Although a multi-sectoral approach was implemented to engage COVID-19 controls at the schools in each country, the organization at local government should also be strengthened to implement school health according to the formulated policy to meet the next public health crisis. The present study highlighted the necessity of formulating a school health policy based on the concepts of PPR that can be associated with the commonality of factors in the three countries provided by the decentralized education. One effective approach would be to strengthen PPR without creating separate PPR strategies for infectious disease control and disaster preparedness.

## Data Availability

Not applicable.

## Abbreviations

CDC: Centers for Disease Control and Prevention
COVID-19: Coronavirus disease 2019
DHSA: Department of Health and Social Affairs
DOE: Department of Education
DRR: Disaster risk reduction
FSM: Federated States of Micronesia
HPS: Health promoting school
KIIs: Key informant interviews
MOE: Ministry of Education
MOH: Ministry of Health
MOHHS: Ministry of Health and Human Services
MOU: Memorandum of understanding
NCDs: Non-communicable diseases
NDOE: National Department of Education
PPR: Prevention, Preparedness, and Response
RMI: Republic of the Marshall Islands
ROP: Republic of Palau
SIDS: Small Islands Developing States
US: United States
WHO: World Health Organization
